# How Has the Availability of Snus Influenced Cigarette Smoking in Norway?

**DOI:** 10.3390/ijerph111111705

**Published:** 2014-11-13

**Authors:** Ingeborg Lund, Karl Erik Lund

**Affiliations:** The Norwegian Institute for Alcohol and Drug Research (SIRUS), P.O. Box 565, Sentrum, 0105 Oslo, Norway; E-Mail: il@sirus.no

**Keywords:** smokeless tobacco, snus, e-cigarettes, smoking

## Abstract

*Background*: In Norway, low-nitrosamine smokeless tobacco (snus) is allowed to compete with cigarettes for market share. We aimed to study how the availability of snus influenced overall tobacco consumption, smoking initiation and smoking cessation. We discuss whether the Norwegian experience with snus can have any transfer value for e-cigarettes. *Methods*: We analysed consumption data from registered and unregistered supply sources of tobacco. We calculated quit-smoking ratios across snus use status in nine datasets comprising a total of 19,269 ever-smokers. Trends in snus use and smoking were derived from time-series of annual; cross-sectional; nationally representative surveys for the period 1985–2013. *Results*: The market share for snus increased from 4% in 1985 to 28% in 2012, but overall tobacco consumption decreased by 20.3% over this same period. Snus was the most common method for smoking cessation. Compared with smokers with no experience of using snus, the quit ratio for smoking was significantly higher for daily snus users in seven of the nine datasets analysed. Among young male adults, the prevalence of smoking (daily + occasional) was reduced from 50% in 1985 to 21% in 2013. Over the same period, use of snus increased from 9% to 33%. This negative correlation (*r* = −0.900, *p* < 0.001) was also observed among young females (*r* = −0.811, *p* < 0.001), but the trend shift in tobacco preferences occurred some years later. *Conclusions*: The experience with snus in Norway might indicate what will happen when alternative nicotine products––are allowed to compete with cigarettes in the nicotine market.

## 1. Introduction

The long-time availability of low-nitrosamine smokeless tobacco (snus) in some Scandinavian countries, including Norway and Sweden, may serve as an example of what could happen on the nicotine market if a low-risk nicotine/tobacco product outside the pharmacological product arsenal (such as e-cigarettes) is allowed to compete with cigarettes. Like e-cigarettes [1], snus is perceived as a less risky alternative to cigarettes [2], but scientists have assessed the relative risk between snus and cigarettes to be even greater than is generally perceived [3,4]. In the US, snus has typically been marketed to smokers as an expedient method for uptake of nicotine in no-smoking areas, as well as a method to quit or reduce the consumption of cigarettes [5]. In Norway and Sweden, all tobacco advertising has been banned since the mid-1970s. Hence, the uptake of snus is not driven by marketing, but takes place in an epistemological climate where smokers and non-smokers tend to overstate the health risk from snus compared with that from cigarettes [2,6]. Moreover, the Scandinavian health authorities have warned smokers against all kinds of snus use, even as a method for smoking cessation [7]. The typical message has been that snus is not a safe alternative to cigarettes.

Thus, the competitive condition for snus in Norway and Sweden has been quite similar to the present market situation for e-cigarettes in the EU: the Tobacco Product Directive aims to restrict e-cigarette advertising to a minimum [8], and the health authorities in most countries have been opposed to recommending e-cigarettes for smoking cessation [9].

Among health authorities, and in particular within the tobacco control community, there is a concern that nicotine products like snus and e-cigarettes will increase the overall use of tobacco/nicotine, recruit non-smokers who otherwise would have stayed nicotine abstinent, delay cessation among cigarette smokers and lead to multi-product use, and thus jeopardize the potential role these products could play in tobacco harm reduction [10,11,12,13]. Representatives of the scientific community [14,15] have therefore put up a research agenda to assess these and other concerns, and because they function as a natural laboratory for observations on snus use, research from Norway and Sweden has been of particular interest.

The extent and nature of the impact on public health of making snus/e-cigarettes available in new markets will to a large extent depend on two factors: (1) the relative risk of snus/e-cigarette use compared with cigarette smoking, and (2) the relative uptake and patterns of use of snus/e-cigarettes by smokers and non-smokers. In contrast to e-cigarettes—where the risk estimates until now have been based on studies of the chemical analysis of the e-liquid and the exhaled vapour using animal studies, on analysis of acute physiological effects in humans and on self-reports from users [9]—the risk estimates for snus use can also be established from long-term epidemiological data on tobacco related diseases such as cardiovascular disease [16], cancers and respiratory diseases [17].

Nutt *et al*. [4] used a multi-criteria decision analysis model to assess the relative importance of different types of harm related to the use of nicotine-containing products, and concluded that snus only caused 5% as much harm as cigarettes. This was consistent with an estimate put forward by Levy *et al*. [3], who assessed the relative risk applying a Delphi design. Given the medical consensus that snus is approximately 90%–95% less harmful than smoking, the overall effect from snus on public health will then be driven by the balance between its beneficial effect on smoking prevalence and its adverse effects on overall prevalence of tobacco use. With this background, the aim of this article is to illustrate how the availability of snus has: (1) influenced the total tobacco consumption in Norway through its role in (2) smoking initiation and (3) smoking cessation. We will then discuss whether the Norwegian experience of allowing snus to compete with cigarettes can have a transfer value for the entrance of e-cigarettes into the nicotine market.

## 2. Methods

### 2.1. Overall Tobacco Consumption

**Figure 1:**
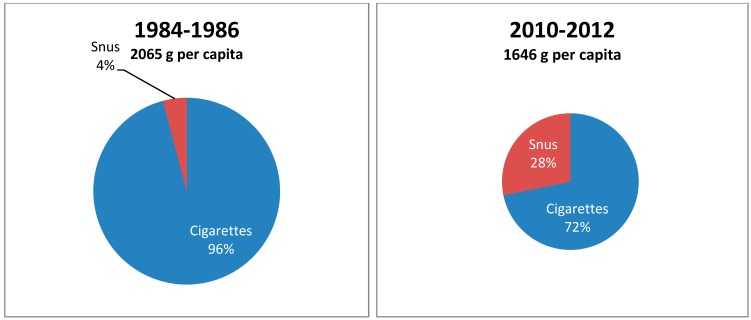
Average annual total tobacco consumption per capita (15+ years) and relative market share of cigarettes and snus in Norway for the periods 1984–1986 and 2010–2012.

Source: Norwegian Directorate of Customs and Excise and Norwegian Institute for Alcohol and Drug Research.

Domestic annual sales of tobacco have been recorded by the Norwegian Directorate of Customs and Excise. Sales of snus and tobacco for hand-rolled cigarettes have been reported by weight, while manufactured cigarettes were converted to weight from the registered number sold (one cigarette equals one gram). Estimates of the magnitude of border trade and travellers’ tobacco imports [18,19] were added to the registered sales. To make consumption data more robust in Figure 1, the means for two three-year periods (1984–1986 and 2010–2013) were compared. To correct for population growth over this period, the total annual consumption was divided by the number of persons above 15 years of age.

### 2.2. Use of Snus in Quit-Smoking Attempts

**Figure 2:**
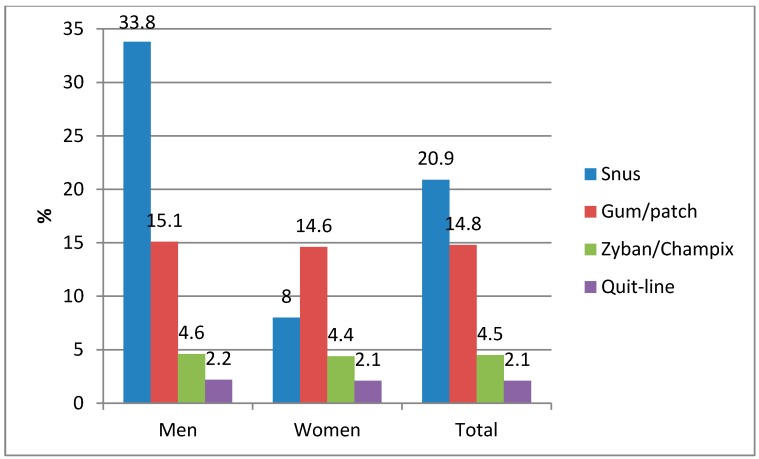
Methods used (alone or in combination) in smoking cessation by Norwegian current (failed quitters) and former daily smokers. Pooled data for 2007–2013.

Source: Statistics Norway and Norwegian Institute for Alcohol and Drug Research.

The study population in Figure 2 included 1830 people who had ever been daily smokers of both genders in the age group 15–50 years. The subjects were identified from a data pool from yearly representative surveys of tobacco behaviour carried out by Statistics Norway by telephone for the years 2007–2013, and included 4887 respondents in total. The mean response rate for the period was 67%. The sampling procedures for these surveys have been described previously [6]. Former daily smokers (*N* = 997) and current daily smokers who reported an attempt to quit smoking (*N* = 639) were asked: “Did you use some of these methods when you last tried to quit smoking (multiple answers possible)?” Response categories were nicotine gum/nicotine patch, snus, Zyban (Bupropion)/Champix (Vareniclin) and “calling the quit line”.

### 2.3. Quit Ratio for Smoking

The quit ratio for smoking is an expression of the number of former daily smokers as a proportion of the total number of people who have ever smoked daily in a population. It is a statistical measure of smoking cessation activity that is recommended for use in tobacco behaviour research [20]. To identify significant differences in the quit ratio for smoking between daily snus users and people who have never used snus, 95% confidence intervals and *p*-values were calculated and are shown in Table 1.

The material and methods for Surveys 1–7 in Table 1 have been described previously [21]. As snus use until recently has been a predominantly male phenomenon in Norway, no gender-specific results were presented in those surveys. Table 1 includes observations from two recent surveys (Surveys 8 and 9 in Table 1 ($Quit\;ratio=\frac{\text{Former smokers x }100}{\text{Ever smokers}}$)). In these samples, sufficient females were using snus to allow gender-specific results. The study population in Survey 8 is identical to the data pool used for the construction of Figure 2 (see above). The study population in Survey 9 included 5813 smokers and former smokers of both genders in the age group 15–80 years surveyed in 2009, 2011 and 2013 in the *Norwegian Monitor Survey* by IPSOS/Synovate. The sampling procedures for this national representative survey have been described previously [22].

**Table 1:** Unadjusted quit ratio for daily smoking (with 95% confidence intervals) according to snus use status in nine Norwegian surveys comprising 14,761 male and 4508 female ever-smokers.

Survey	Year	Number of Ever-Smokers	Age Group	Daily Snus User	Never Used Snus	*p*-value
**Males**						
1	2003–2008	3604	16–74	80.4 (74.9–85.9)	51.9 (50.1–53.7)	< 0.001
2	2007	423	16–20	54.8 (45.2–64.9)	22.9 (17.4–28.4)	< 0.001
3	2006	458	19–30	81.1 (68.5–93.7)	62.7 (56.9–68.4)	0.010
4	2007	2016	15–91	61.6 (53.5–69.7)	52.5 (50.2–54.8)	0.035
5	2006	729	21–30	75.4 (65.2–85.6)	44.9 (40.2–49.6)	< 0.001
6	2006	639	21–30	89.7 (81.9–97.5)	50.0 (45.1–54.9)	< 0.001
7	2007	2572	20–50	72.7 (81.9–97.5)	43.3 (40.6–46.0)	< 0.001
8	2009–2013	1543	15–74	80.8 (75.4–86.2)	55.7 (52.9–58.5)	< 0.001
9	2009–2013	2777	15–80	74,6 (69.8–79.4)	65.5 (63.6–67.5)	< 0.001
Total	--	14,761	Weighted mean	74.8 (72.8–76.8)	52.3 (51.4–53.2)	< 0.001
**Females**						
8	2009–13	1472	15–74	85.7 (75.0–95.2)	52.0 (49.4–54.6)	< 0.001
9	2009–13	3036	15–80	74.8 (65.8–83.8)	61.6 (59.8–63.4)	< 0.001
Total	--	4508	Weighted mean	78.4 (71.5–85.3)	56.1 (54.6–57.6)	< 0.001

### 2.4. Smoking Initiation

**Figure 3:**
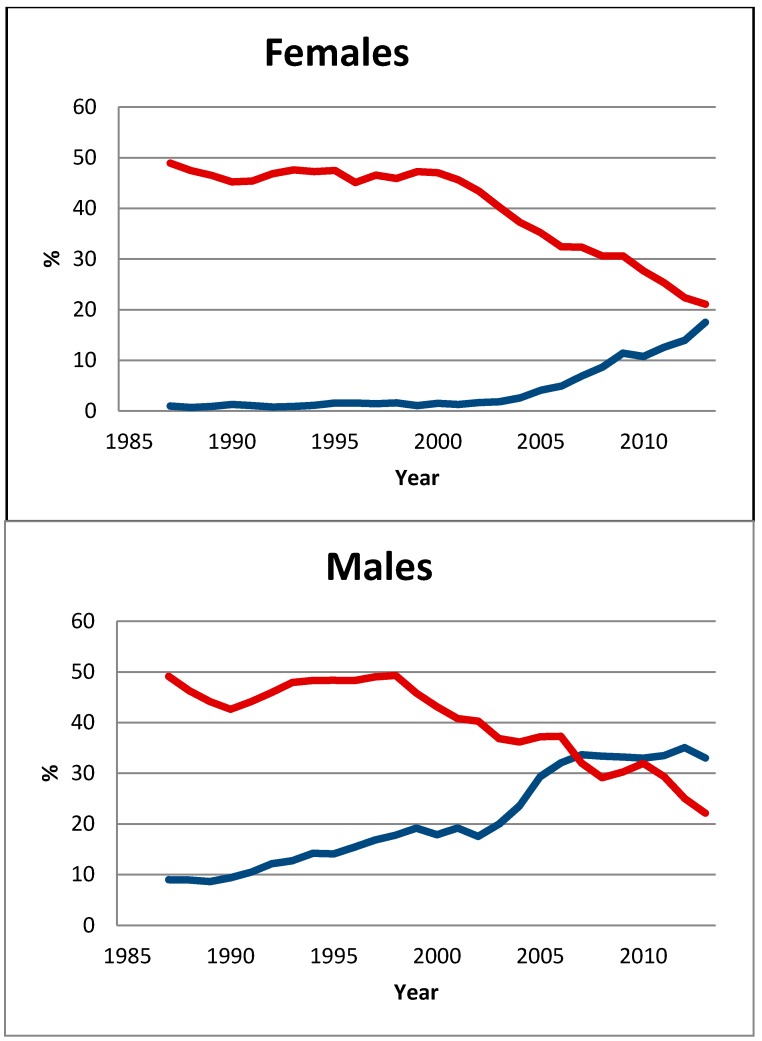
Use (daily and occasional) of snus (blue line) and cigarettes (red line) by Norwegian males and females in the age group 16–30 years for the period 1985–2013. Three-year moving averages.

Source: Statistics Norway and Norwegian Institute for Alcohol and Drug Research.

As the initiation of cigarette smoking is negligible after the age of 30 years, the prevalence of smokers (daily and occasional) in the age group 15–30 years was used as a proxy for smoking uptake [20]. Data for this age group were derived from a subset sample of the yearly cross-sectional surveys carried out by Statistics Norway, described in connection with the construction of Figure 2. Trend estimates for cigarette smoking and snus use in Figure 3 were based on three-year moving averages for the period 1985–2013.

## 3. Results

The annual overall per capita (15+ years) consumption of tobacco was 2065 grams for the years 1984–1986. In this period, 96% of the tobacco was consumed as cigarettes (manufactured or roll-your-own), while the market share for snus was only 4%. In the period 2010–2012 the market share for snus had increased to 28%, while cigarettes comprised 72% of the tobacco market. Over these three decades, the overall consumption of tobacco in Norway (registered + unregistered) declined by 20.3% to 1646 grams per capita in 2010–2012.

Among Norwegian ever-daily smokers who had used a specific method to quit smoking, snus was the most common (20.9%), followed by pharmacological nicotine products (14.8%) (Figure 2). The use of snus to quit smoking was particularly prevalent among male quitters (33.8%), while pharmacological nicotine was the most common method among females (14.6%).

Compared with smokers with no experience of using snus, the quit ratio for smoking among males was significantly higher for daily snus users in seven of the nine datasets in Table 1. The weighted mean for quit-smoking ratios across all datasets was 74.8 (CI 72.8–76.8) among male daily snus users and 52.3 (CI 51.4–53.2) among male non-users of snus. Among females, the same difference in the quit ratio for smoking was observed between daily users [78.4 (CI 71.5–85.3)] and non-users of snus [56.1 (54.6–57.6)].

Among young male adults, the prevalence of smoking (daily + occasional) decreased from 50% in 1985 to 21% in 2013. Over the same period, the prevalence of snus use increased from 9% to 33%. Around 2008, snus surpassed cigarettes as the most popular nicotine product. The negative correlation between snus use and cigarette smoking among males in this age group (*r* = −0.900, *p* < 0.001) was also observed among females (*r* = −0.811, *p* < 0.001). However, compared with that of males, the trend shift in tobacco preferences among women seems to have occurred some years later.

## 4. Discussion

The introduction of low-nitrosamine snus to the nicotine market in the 1990s was followed by an increase in snus use. This was first observed among men, but has recently also occurred in women. However, the increased use of snus has not led to an increase in overall tobacco consumption, as sales of cigarettes have decreased (Figure 1). Three observations lend support to the hypothesis that the strong inverse association between snus use and cigarette smoking might be causal.

First, snus seems to play a significant role in smoking cessation. Use of snus is reported by ever-smokers to be the preferred method for quitting (Figure 2), and in several studies from Norway [23,24] and Sweden [25,26,27] former smokers are seen to make up the largest segment of snus users. Moreover, daily snus use seems to be associated with particularly high quit rates for smoking (Table 1), as also observed in studies from Sweden [25,26,27,28,29,30,31,32]. Several randomized controlled trials have also demonstrated that use of smokeless tobacco increases the likelihood of quitting smoking [33,34,35,36,37,38,39]. Not only does snus surpass the use of pharmacological nicotine products in quit-smoking attempts, some studies also indicate that snus might be more effective than pharmacological nicotine products in smoking cessation [23,40]. The enhanced effect from snus over medicinal nicotine products (efficacy) combined with the high acceptance of snus in smoking cessation implies that the impact on smoking abstinence at the population level (effectiveness) is much higher for snus than for other products.

A second mechanism for a causal relationship between increased market share for snus and the reduced market share for cigarettes could be that snus attracts tobacco-prone youth who otherwise would have started to smoke. Of course, we do not know the counterfactual—what the rate of decline in smoking among youth would have been without the availability of snus over the same time frame. However, our hypothesis of a relationship is supported because a large segment of the young snus users have characteristics that usually predict uptake of cigarette smoking [41,42,43]. Moreover, the hypothesis is also supported by the simultaneous trend shift for smoking and snus use among young adults, and the fact that this has occurred in both sexes at different times (Figure 3). These coincidental trend shifts in tobacco preferences haves also been observed in other datasets in Norway [41,44,45]. As far as we know, the rapidity of the decline in smoking among youth in Norway has not been matched in any other Western country.

A third mechanism through which snus may have contributed to a decrease in cigarette consumption is that snus is used by many smokers to reduce smoking intensity or as a nicotine replacement where smoking is prohibited [21,23,24]. In Norway, dual users of snus and cigarettes have a weekly consumption of cigarettes that is 40% below that of exclusive smokers [24]. However, the proportion of dual users of snus and cigarettes has been quite small, and has not increased during the period when snus became more popular [24].

Even if snus could be classified as neither a *sufficient* condition nor a *necessary* condition to reduce smoking in a given society, the experience from Norway indicates that snus should be considered as an important *contributory* factor in the decline of smoking. However, it is important to emphasize that the observed marked shift from cigarettes to snus in Norway could have been even stronger if misperceptions of the relative risk of the two products had been adjusted in accordance with medical consensus. Smokers [2,6,46] and even GPs [47] tend to overestimate the health risk from snus use compared with that from cigarette smoking, and among smokers who incorrectly perceive snus as being as risky as cigarettes, the willingness to try snus in an attempt to quit smoking is quite low [2]. Willingness to use snus in quit-smoking attempts is significantly higher for smokers who, consistent with scientific evidence, believe that the health risks are far lower for snus than for cigarettes [2]. Thus, dissemination of information from authorities to correct misconceptions of relative risk, as recommended by some US researchers [48], might speed up the trajectory from cigarettes to snus to quitting completely.

### Transfer Value to E-Cigarettes

In Norway, snus has emerged as a realistic alternative to conventional cigarettes because of its ability to deliver nicotine without the combustion and the toxicants in tobacco smoke, the fact that snus can be used in smoke-free places, the competitive price and the perceived potential for harm reduction (even if this potential is under-estimated). The new snus products that were introduced to the nicotine market in the 1990s differed from conventional smokeless tobacco in that they were lower in major carcinogens such as tobacco-specific nitrosamines and polycyclic aromatic hydrocarbons [49,50], did not require spitting, came in a variety of flavours such as mint and eucalyptus, and were presented in small pouches packed in elegant and colourful tin boxes. These innovations certainly made snus more user-friendly and probably also increased its appeal, not only to established smokers, but also to young people with no prior history of tobacco use. However, snus samples from the US and India has been identified with nitrosamines as high as traditional moist snuff [51,52], so within the product category there are important variations.

E-cigarettes share many of the above abilities to compete with cigarettes on the nicotine market (price, product variability, packaging, perceived harm reduction, identity formation and social and symbolic functions) [53]. Furthermore, e-cigarettes also offer some important advantages over snus. The repetitive hand-to-mouth motion, the visual cue of smoke-like vapour and the cigarette-like nicotine delivery device can maintain the behavioural component of smoking and the identity that many smokers appreciate. In markets where both products have access to the nicotine market, e-cigarettes have generally achieved higher popularity than snus [54]. Studies have also shown that smokers have reported a greater liking for e-cigarettes [55,56,57] than for snus [54,58,59]. Thus, the high receptivity towards snus in Scandinavia may not exist in other cultures. In the US current use of snus in women is almost non-existing and the potential impact on smoking cessation associated with snus use might not be replicable in the US [60].

On the other hand, many jurisdictions have recently banned or restricted indoor vaping, while the use of snus seems to remain more or less unregulated. Moreover, the customer base for e-cigarettes seems to be strictly limited to smokers, while snus has also had some appeal to a segment of non-smokers, who comprise the majority of the population. However, e-cigarettes seem to appeal to both sexes, while snus use for the most part is observed among males.

All in all, e-cigarettes possess most of the characteristics that made snus a significant competitor with cigarettes on the nicotine market in Norway. Moreover, e-cigarettes have unique behavioural and social functions beyond those of snus, resulting in higher popularity. Further, the customer base for e-cigarettes is not gender-specific (as it is for snus), and is therefore much larger. Consequently, at least in countries where the market conditions for e-cigarettes are similar to those for snus in Norway, the experience with snus may be replicated for e-cigarettes. Most probably, given their competitive advantages over snus, e-cigarettes would compete even better with cigarettes than snus when introduced to new markets.

## 5. Limitations

In principle, the contrasting of quit ratios for smoking between daily snus users and never snus users (Table 1), should be adjusted for variables known to influence smoking cessation. The results presented here are gender specific, and in some of the nine surveys restricted to specific age groups. However, length of education, risk perceptions and a full range of smoking behavior components (such as smoking intensity) are also predictors of successful smoking cessation attempts and thereby possible confounders. Several studies has shown that dual users of snus and cigarettes consume fewer cigarettes than do exclusive smokers [24], and that nicotine substitution with snus is the reason [21,23]. We had no information on cigarette consumption at the point in time where the smokers who later were to become dual users still was using cigarettes exclusively. Therefore, we were unable to adjust for smoking intensity. However, we have no evidence that low-intensity-daily-smokers are particularly attracted to snus (selection bias).

## 6. Conclusions

In Norway, snus has contributed to a decrease in cigarette consumption through three mechanisms: (1) as a method of smoking cessation, (2) as an alternative product for new generations of tobacco-prone youth who otherwise would take up smoking, and (3) as an alternative to cigarettes for smokers who are unwilling or unable to quit smoking altogether. The availability of snus is neither a sufficient nor a necessary condition for a reduction in tobacco smoking. Tobacco control measures have perhaps been the most important factor in smoking reduction. However, in countries that already have a robust infrastructure for tobacco control, tobacco harm reduction strategies might be a necessary supplement to reduce smoking related mortality. Through the same mechanisms as with snus, e-cigarettes might appear as an important contributory factor towards a decrease in smoking. However, the effect would very much depend on how market access is regulated.
